# Evaluation of the Use of Phenobarbital and Benzodiazepines in the Management of Alcohol Withdrawal Syndrome in Patients Requiring Neurological/Neurosurgical Critical Care: A Propensity-Matched Analysis

**DOI:** 10.7759/cureus.61952

**Published:** 2024-06-08

**Authors:** Melanie Norris, Hannah Mak, Christine T Fong, Andrew M Walters, Cuong V Hoang, Abhijit V Lele

**Affiliations:** 1 Pharmacy, Harborview Medical Center, Seattle, USA; 2 Anesthesiology and Pain Medicine, University of Washington, Harborview Medical Center, Seattle, USA; 3 Anesthesiology and Pain Medicine, Harborview Medical Center, Seattle, USA

**Keywords:** ciwa, alcohol withdrawal, critical care, neuro, benzodiazepines, barbiturate

## Abstract

Objective

There is growing interest in the use of phenobarbital for alcohol withdrawal syndrome in critically ill patients, though experience in neurologically injured patients is limited. The purpose of this study was to compare the safety and effectiveness of phenobarbital-containing alcohol withdrawal regimens versus benzodiazepine monotherapy in the neurocritical care unit.

Methods

We conducted a retrospective cohort study of adult patients admitted to the neurocritical care unit from January 2014 through November 2021 who received pharmacologic treatment for alcohol withdrawal. Treatment groups were defined as benzodiazepine monotherapy versus phenobarbital alone or in combination with benzodiazepines. The primary outcome was the percentage of patients requiring intubation after receiving alcohol withdrawal treatment. Secondary outcomes included all-cause, in-hospital mortality, intensive care unit length of stay, discharge disposition, change in Glasgow Coma Scale (GCS) score, and the use of adjunctive agents.

Results

We analyzed data from 156 patients, with 77 (49%) in the benzodiazepine group and 79 (51%) in the phenobarbital combination group. The groups were well-balanced for baseline characteristics, though more males (67, 85%) were in the phenobarbital group. Only three (1.9%) patients received phenobarbital monotherapy, and the rest (153, 98.1%) received combination therapy. The percentage of patients requiring mechanical ventilation was significantly higher in the phenobarbital combination group compared to benzodiazepine monotherapy (39% (n=31) versus 13% (n=10); OR: 4.33, 95% CI: 1.94-9.66; p<0.001). The use of adjunctive propofol and dexmedetomidine was higher in the phenobarbital group (propofol 35% (n= 28) versus 9% (n=7) and dexmedetomidine 30% (n=24) versus 5% (n=4), respectively). Patients in the phenobarbital group also had lower GCS scores and higher Clinical Institute Withdrawal Assessment of Alcohol (CIWA-Ar) scores during their intensive care unit admission, possibly suggesting more severe alcohol withdrawal. There was no difference in intensive care unit length of stay, all-cause, in-hospital mortality, discharge disposition, or therapeutic adjuncts.

Conclusions

Combination therapy of phenobarbital plus benzodiazepines was associated with higher odds of requiring mechanical ventilation. Few patients received phenobarbital monotherapy. Additional studies are needed to better compare the effects of phenobarbital monotherapy versus benzodiazepines in neurocritical patients.

## Introduction

Alcohol withdrawal syndrome (AWS) is a complex constellation of symptoms that manifests as a consequence of abrupt reduction or cessation in alcohol intake after chronic use. These symptoms can range from mild tremors and agitation to more severe symptoms of hallucinations, seizures, or delirium tremens (DTs). Severe AWS remains a significant contributor to morbidity and in-hospital mortality [1,2].

The pathophysiology of AWS is related to neuronal hyperexcitation due to the dysregulation of excitatory (dopamine, norepinephrine, and N-methyl-D-aspartate (NMDA)) receptors and inhibitory (alpha-2 and gamma-aminobutyric acid (GABA)-A) receptors that develop after chronic alcohol ingestion. The primary agents used for AWS management are targeted to activate the GABA pathway and to reduce NMDA and glutamate activity. Benzodiazepines (BZDs) are considered the primary treatment for AWS through their mechanism of increasing GABA transmission. BZDs effectively control symptoms, reduce alcohol withdrawal seizures, and prevent DTs [2]. More recently, phenobarbital (PHB) has been used successfully as an adjunct to BZD therapy or in place of BZD therapy for treating BZD-refractory AWS [1-5]. One significant concern with these therapies is the risk of excessive sedation, which may confound the neurologic examination, particularly in patients requiring neurological/neurosurgical critical care (hereby referred to as NCC). Thus, AWS management in this population requires balancing symptoms and preserving the neurological exam.

Identification of AWS in NCC is challenging, as many patients are unable to provide a reliable history due to their already depressed neurological exam at the time of ictus. It may be difficult to distinguish whether autonomic excitation or seizures are a manifestation of AWS or a symptom of the primary neurological injury [1]. Furthermore, patients with primary neurological diagnoses have often been excluded from larger studies about AWS management. The purpose of this study was to characterize the regimens used for AWS management at our institution and to determine the safety and effectiveness of PHB-containing AWS regimens versus BZD monotherapy in NCC.

## Materials and methods

Study design and patient population

This retrospective cohort study was conducted at Harborview Medical Center (HMC), a 413-bed county hospital and level-one trauma center. The study (STUDY 00014298) was approved (14 February 2022) by the University of Washington Medical Center Institutional Review Board (IRB), and a waiver of informed consent was in place. Patients 18 years and older and who were admitted to the Neurocritical Care Service (NCCS) from January 1, 2014, through November 30, 2021, were included in the study if they were treated for confirmed or suspected AWS during the encounter. Patients who were receiving mechanical ventilation before AWS treatment initiation or those with a history of benzodiazepine or other illicit drug use were excluded. Patients with an admitting diagnosis of seizures or status epilepticus were also excluded to avoid confounding the benzodiazepine indication.

Data collection

Baseline patient characteristics, including age, sex, admitting diagnosis, and blood alcohol level, were extracted from the database. Baseline or time zero was defined as the time when the first AWS treatment drug was administered. The severity of AWS prior to treatment initiation was assessed by the Clinical Institute Withdrawal Assessment of Alcohol (CIWA-Ar) score [1,6]. The Glasgow Coma Scale (GCS) was used to assess neurologic function and was collected as the value prior to AWS treatment initiation (baseline), the minimum or worst score during the intensive care unit (ICU) admission, and the score upon discharge from the hospital. Exposure to AWS treatment drugs in milligrams was abstracted through drug dispensing reports [2].

Outcomes

The primary outcome was the percentage of patients requiring intubation after receiving phenobarbital (either alone or in combination with benzodiazepines) versus benzodiazepine monotherapy for AWS management. The secondary outcomes of this study were to determine whether use of phenobarbital-containing regimens versus benzodiazepines alone was associated with differences in all-cause mortality, ICU length of stay, discharge disposition, change in GCS, and use of adjunctive agents (including additional sedatives or antipsychotics).

Statistical analysis

Due to the significant difference in size between the benzodiazepine monotherapy group and the phenobarbital combination group, propensity score matching based on the primary cause of neurologic injury and baseline GCS was used to create the final cohort for statistical analysis. We compared the baseline characteristics between the two groups, using proportions for categorical variables and medians for continuous variables. Differences between the two groups were assessed using chi-square tests for categorical variables and the Mann-Whitney U test for continuous variables. Microsoft Excel (Microsoft® Corp., Redmond, WA) and GraphPad Prism software (GraphPad Software, San Diego, CA) were used.

## Results

Derivation of the study cohort 

**Figure 1:**
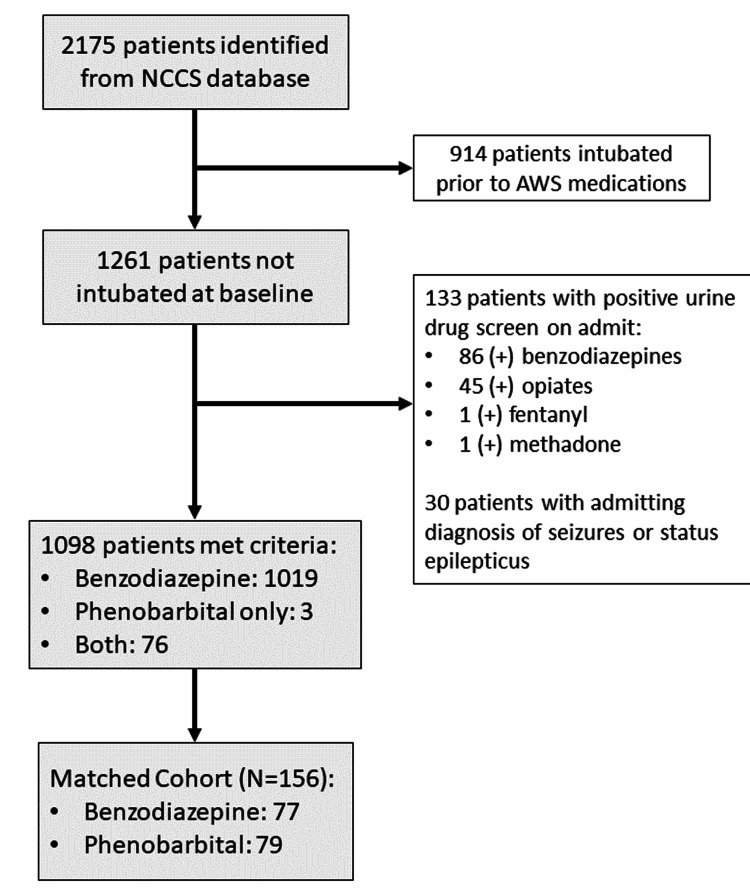
Flow diagram of cohort selection NCCS: neurocritical care service; AWS: alcohol withdrawal syndrome

Between January 2014 and November 2021, a total of 2,175 patients were treated for confirmed or suspected AWS. Specifically, 914 patients were excluded for being intubated before receiving AWS treatment, and 133 were excluded for a positive urine drug screen on admission. A further 30 patients were excluded due to an admitting diagnosis of status epilepticus. A total of 1,098 patients met our enrollment criteria, with 1,019 (93%) receiving benzodiazepine monotherapy. Propensity score matching yielded a cohort of 77 (49%) patients in the benzodiazepine monotherapy group and 79 (51%) patients in the phenobarbital combination group. Only three patients (4%) received phenobarbital monotherapy; most patients in the phenobarbital group received combination therapy with benzodiazepines and phenobarbital (Figure 1).

Cohort characteristics

Baseline characteristics are outlined in Table 1. The two groups were well-balanced regarding baseline characteristics, though there was a higher proportion of male patients in the combination therapy group. The mean age was 53.5 years (SD: 13.15), and the most common admitting diagnosis was traumatic brain injury (38.5%, n=60), followed by spontaneous intracerebral hemorrhage (18%, n=28) and acute ischemic stroke (16.7%, n=26). The median baseline GCS for this cohort was 14 (IQR: 13-15).

**Table 1:** Characteristics of a propensity-matched cohort of patients requiring neurological/neurosurgical critical care by type of alcohol withdrawal regimen SD, standard deviation; ICH, intracerebral hemorrhage; GCS, Glasgow Coma Scale; IQR, interquartile range; CIWA-Ar, Clinical Institute Withdrawal Assessment of Alcohol

	Benzodiazepine Group (n=77)	Phenobarbital Group (n=79)	P-value
Age, mean (SD)	53.5 (13.54)	53.6 (12.85)	0.811
Male sex, n (%)	43 (56%)	67 (85%)	p<0.001
Primary Diagnosis, n (%)			
Traumatic brain injury	29 (38%)	31 (39%)	0.839
Spontaneous ICH	14 (18%)	14 (18%)	0.940
Acute ischemic stroke	13 (17%)	13 (16%)	0.943
Other	10 (13%)	11 (14%)	0.864
GCS, Median (IQR)			
Admission GCS score	15 (13-15)	14 (12.5-15)	0.632
Worst GCS score	13 (9-14)	9 (5-13)	p<0.001
Discharge GCS score	15 (14-15)	15 (15-15)	0.465
CIWA-Ar Score, Median (IQR)			
First CIWA-Ar score	7.5 (4-11)	9 (5-16)	0.054
Highest CIWA-Ar score	14 (7-19)	23 (18-27)	p<0.001

**Figure 2:**
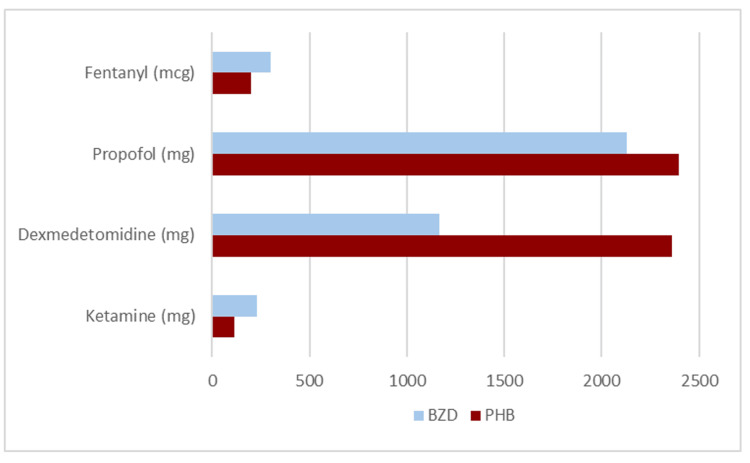
Median exposure to adjunctive agents during intensive care unit admission BZD: Benzodiazepine, PB: Phenobarbital

The median cumulative lorazepam dose was greater in the phenobarbital combination group compared to the benzodiazepine group (17 mg (IQR: 6-40) versus 4 mg (IQR: 2-11), respectively; p<0.001). The median cumulative phenobarbital dose was 260 mg ((IQR: 130-615), range: 0-2,448 mg). A higher proportion of patients in the phenobarbital group received adjunctive sedatives compared to the benzodiazepine group: propofol 35% (n=28) versus 9% (n=7) (OR: 5.49 (95% CI: 2.22-13.55); p<0.001) and dexmedetomidine 30% (n=24) versus 5% (n=4) (OR: 7.96 (95% CI: 2.61-24.28); p<0.001), respectively. The use of fentanyl and antipsychotics was similar between the two groups (Table 2 and Figure 2).

**Table 2:** Exposure to adjunctive agents during intensive care unit admission Adjunctive use of antipsychotic agents included the administration of haloperidol, quetiapine, or olanzapine. A significantly higher proportion of patients in the phenobarbital group received propofol compared to the benzodiazepine group (OR: 5.49 (95% CI: 2.22-13.55); p<0.001). The proportion of patients requiring dexmedetomidine was also higher in the phenobarbital group (OR: 7.96 (95% CI: 2.61-24.28); p<0.001).

Number (%)​	Benzodiazepine Group (n=77)​	Phenobarbital Group (n=79)​	P-value
Fentanyl​	31 (40%)	37 (47%)	0.408
Propofol	7 (9%)	28 (35%)	p<0.001
Dexmedetomidine​	4 (5%)	24 (30%)	p<0.001
Ketamine​	2 (2.6%)	4 (5%)	0.423
Antipsychotics​	24 (31%)	35 (44%)	0.091

A total of 31 patients (39%) in the phenobarbital combination therapy group required mechanical ventilation after AWS treatment had been initiated, as compared to 10 patients (13%) in the benzodiazepine monotherapy group (OR: 4.33 (95% CI: 1.94-9.66); p<0.001). The median intensive care unit length of stay was similar between the phenobarbital and benzodiazepine groups (three days (IQR: 2-6) versus two days (IQR: 2-6), respectively; p=0.065). Differences in in-hospital mortality were insignificant (5% versus 6.5%, respectively; p=0.702). Most patients were able to discharge home (54% in the phenobarbital group versus 56% in the benzodiazepine group; p=0.859).

Median baseline CIWA-Ar scores were similar between the two groups (9 (IQR: 5-16) versus 7.5 (IQR: 4-11); p=0.054), but the worst CIWA-Ar scores recorded during intensive care unit admission were higher in the phenobarbital group (median: 23 (IQR: 18-27) versus 14 (IQR 7-19), respectively; p<0.001). A similar trend was observed in GCS, with the worst GCS being lower in the phenobarbital group compared to the benzodiazepine group (median: 9 (IQR: 5-13) versus 13 (IQR 9-14), respectively; p<0.001). Despite this trend, the median GCS at the time of discharge was 15 in both groups (Table 3).

**Table 3:** Primary and secondary outcomes between benzodiazepine and phenobarbital groups AWS, alcohol withdrawal syndrome; ICU, intensive care unit; IQR, interquartile range

	Benzodiazepine Group (n=77)​	Phenobarbital Group (n=79)​	Between Group Comparison
Primary Outcome			
Patients requiring mechanical ventilation after initiation of AWS treatment, no (%)	10 (13%)	31 (39%)	OR: 4.33 (95% CI:1.94-9.66); p<0.001
Secondary Outcomes			
ICU length of stay, median (IQR)	2 days (2-6)	3 days (2-6)	p=0.065
In-hospital mortality, no (%)	5 (6.5%)	4 (5%)	p=0.702
Discharge Disposition, no (%)			
Home	43 (56%)	43 (54%)	p=0.859
Skilled nursing facility	14 (18%)	13 (16%)	p=0.776
Rehabilitation	4 (5%)	11 (14%)	p=0.064
Other	11 (14%)	8 (10%)	p=0.427

## Discussion

In this single-institution retrospective cohort study of neurocritical care patients, the combination use of benzodiazepines and phenobarbital was associated with higher odds of requiring mechanical ventilation compared to benzodiazepine monotherapy. Patients who were exposed to phenobarbital tended to receive higher cumulative medication dosages and more adjunctive sedative agents during their intensive care unit stay. The increased exposure to propofol and dexmedetomidine observed in the combination group closely aligned with the mechanical ventilation rate. Despite the increased rate of intubation, there was no significant difference with regard to intensive care unit length of stay, discharge disposition, or all-cause mortality.

Throughout their intensive care unit admission, patients in the combination group had higher recorded CIWA-Ar scores than the benzodiazepine monotherapy group. Those in the combination group also had higher cumulative benzodiazepine exposure than the monotherapy group. In several patients, phenobarbital was used as an adjunct after multiple doses of benzodiazepines had failed to adequately control symptoms. Thus, it is possible the increased incidence of mechanical ventilation observed in the combination group represents a bias of patients with more severe AWS, who may have been at greater risk of respiratory complications irrespective of initial drug therapy.

There is growing evidence supporting the use of phenobarbital for the management of AWS in critically ill patients, though experience in neurologically injured patients remains limited [1,2,4,7,8]. Oks et al. [9] evaluated phenobarbital use in medical ICU patients and administered 130 mg of phenobarbital every 15 minutes until the target Richmond Agitation Sedation Scale (RASS) was achieved (mean cumulative dose: 2000 mg). In their cohort of 86 patients, 17 patients (20%) required mechanical ventilation after AWS treatment initiation, though no intubation event was found to be directly correlated to phenobarbital administration [9]. Similar to our study, Nguyen et al. [10] compared combination therapy with symptom-triggered lorazepam plus phenobarbital (mean cumulative dose: 500 mg) versus lorazepam monotherapy in 72 patients requiring ICU admission for severe AWS. They observed a numerically higher number of intubations in the combination group, three patients (8.3%) versus zero patients in the monotherapy group, though this difference was not statistically significant [10]. Both studies found similar clinical outcomes and no significant increase in adverse events when phenobarbital was used for AWS.

More recently, Alwakeel et al. described the results of a pre- and post-protocol study comparing phenobarbital versus lorazepam for 102 adults who required medical ICU admission for severe AWS. Phenobarbital was given in 130 mg doses every 15-30 minutes up to 15 mg/kg ideal body weight (median dose: 500 mg). Patients with other indications for ICU admission, besides AWS, were excluded. The phenobarbital protocol was associated with reduced intensive care unit length of stay, lower rate of intubation, and fewer adjunctive agents needed [11]. Malone et al. also used a phenobarbital loading dose strategy, followed by an oral taper (median cumulative dose: 800 mg). In their cohort of 147 adult patients, phenobarbital was associated with a significant reduction in pulmonary complications compared to benzodiazepines [12].

Our results differ from these other studies [7,13-15] in that the percentage of patients requiring mechanical ventilation was higher, particularly in the phenobarbital combination group. Interestingly, the medication doses (both phenobarbital and lorazepam) received in these studies were much higher than what was observed in our cohort, though the frequency of respiratory complications was reduced in the comparator studies. In most of these studies, the reason for intensive care unit admission was the management of severe AWS, whereas all of our patients were admitted to the intensive care unit for primary neurologic diagnoses. It is likely that the differences in patient population and the underlying disease states leading to intensive care unit admission contributed to the significant difference in intubation rates that we observed.

There are several limitations to consider when interpreting the results of this study. Due to the project's retrospective nature, we could not adjudicate the exact indication for each medication order. Given a portion of our cohort were neurosurgical patients, it is possible some of the intubation events we captured were performed by anesthesia to facilitate procedures. We also relied on surrogate markers for adverse drug reactions and the relationship between AWS treatment initiation. Specific adverse events, such as excess sedation or changes to a neurologic exam, could not be assessed directly. We excluded patients who were receiving mechanical ventilation prior to initiating treatment for AWS and those with positive urine drug screens on admission, which could have introduced bias for selecting patients with less severe alcohol withdrawal. Ideally, AWS treatment strategies would be compared as benzodiazepine monotherapy versus barbiturate monotherapy; however, we were unable to do so due to the high prevalence of combination therapy in this cohort.

Overall, the use of phenobarbital, particularly phenobarbital monotherapy, was lower than anticipated. Our institution has had a phenobarbital for AWS protocol for several years, but it appears that utilization in this patient population is low. An updated phenobarbital protocol was implemented in March 2022, so it is possible phenobarbital utilization may increase in the coming years as providers become more familiar with use in this setting. This protocol emphasizes a phenobarbital loading dose strategy, with either 10 mg/kg or 15 mg/kg loading doses recommended depending on alcohol dependence, AWS severity, and history of complicated AWS. In the present cohort, phenobarbital dosing was inconsistent, and few patients received weight-based loading doses of phenobarbital. It would be beneficial to conduct a similar investigation in a few years to see if there has been an increase in phenobarbital monotherapy after the implementation of this new protocol.

Conducting a retrospective chart review may be more insightful for this clinical question, as it would be easier to investigate why medications were administered (AWS versus unrelated procedures) and determine the timing from AWS treatment to initiation of mechanical ventilation. The present study extracted information from a historical database of patients admitted to NCCS, and while this method was helpful for quickly identifying patients of interest and efficiently pulling large amounts of data, some variables were missing clinical context.

## Conclusions

Our results suggest that combination use of benzodiazepines and phenobarbital for alcohol withdrawal syndrome management in neurocritical care patients may be associated with greater odds of requiring mechanical ventilation. It is important to note that we were unable to compare benzodiazepine monotherapy versus barbiturate monotherapy in our cohort due to low phenobarbital utilization. Additional studies are needed to further evaluate whether there is any meaningful difference in safety between these alcohol withdrawal syndrome treatment strategies in neurologically injured patients.
